# Localization and Directionality of Surface Transport in Bi_2_Te_3_ Ordered 3D Nanonetworks

**DOI:** 10.1021/acsnano.3c04160

**Published:** 2023-07-06

**Authors:** Alejandra Ruiz-Clavijo, Nicolás Pérez, Olga Caballero-Calero, Javier Blanco, Francesca Peiró, Sergi Plana-Ruiz, Miguel López-Haro, Kornelius Nielsch, Marisol Martín-González

**Affiliations:** § Instituto de Micro y Nanotecnología, IMN-CNM, CSIC (CEI UAM+CSIC) Isaac Newton 8, E-28760, Tres Cantos, Madrid, Spain; † Institute for Metallic Materials, IFW-Dresden, Helmholtzstrasse 20, 01069 Dresden, Germany; || LENS-MIND, Department of Electronics and Biomedical Engineering, Universitat de Barcelona, 08028 Barcelona, Spain; ¥ Institute of Nanoscience and Nanotechnology (IN2UB), Universitat de Barcelona, 08028 Barcelona, Spain; ¢ Scientific & Technical Resources, Universitat Rovira i Virgili, 43007 Tarragona, Spain; ‡ Departamento de Ciencia de los Materiales e Ingeniería Metalúrgica y Química Inorgánica, Facultad de Ciencias, Universidad de Cádiz, Cádiz 11510, Spain

**Keywords:** metamaterials, bismuth telluride, 3D-AAO, nanonetwork, surface transport, localization effects

## Abstract

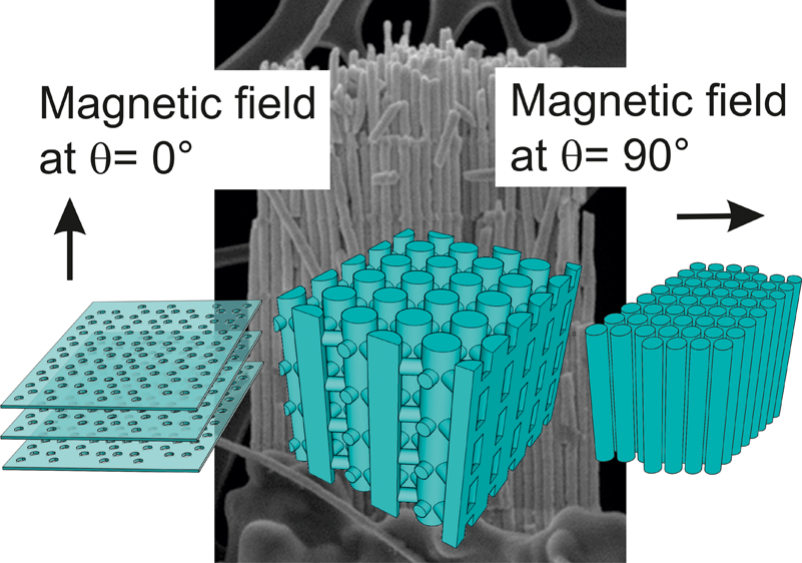

The resistance of an ordered 3D-Bi_2_Te_3_ nanowire nanonetwork was studied at low temperatures. Below 50 K the increase in resistance was found to be compatible with the Anderson model for localization, considering that conduction takes place in individual parallel channels across the whole sample. Angle-dependent magnetoresistance measurements showed a distinctive weak antilocalization characteristic with a double feature that we could associate with transport along two perpendicular directions, dictated by the spatial arrangement of the nanowires. The coherence length obtained from the Hikami–Larkin–Nagaoka model was about 700 nm across transversal nanowires, which corresponded to approximately 10 nanowire junctions. Along the individual nanowires, the coherence length was greatly reduced to about 100 nm. The observed localization effects could be the reason for the enhancement of the Seebeck coefficient observed in the 3D-Bi_2_Te_3_ nanowire nanonetwork compared to individual nanowires.

Nanowires (NWs) are excellent model systems for investigating the effects of low dimensionality in materials.^1−5^ Controlling the diameter of the NW, and hence its surface-to-volume ratio, the presence or relevance of surface-related characteristics can be addressed.^3−9^ This is of particular interest in the case of topological insulators (TIs) owing to electronic surface states (topologically protected) independent from bulk states.^6,8^ Tetradymite chalcogenides Bi_2_Te_3_, Bi_2_Se_3_, and Sb_2_Te_3_ are small band gap semiconductors with wide application in state-of-the-art Peltier cooling devices or thermoelectric power generators.^10^ They are also strong 3D-TIs,^11^ with topologically protected states predicted to appear on any free surface regardless of the crystallographic orientation. Nevertheless, it is worth mentioning that most investigations on Tls are performed on single crystals. A linear dispersion relation attributed to electrons in topologically protected surface states has been measured particularly in Bi_2_Te_3_ using angle-resolved photoemission spectroscopy.^12,13^ Experimental observation of the quantum-Hall effect and the weak antilocalization effect are also typical signatures linked to the presence of topologically protected surface states in tetradymite chalcogenides.^4^ Electrical transport experiments in thin films and individual NWs of Bi_2_Te_3_ have shown deviation from their bulk counterparts with increased mobility and a reduced Seebeck coefficient.^14^ These findings could be explained by a two-channel transport model that accounts for the material’s surface and the bulk. In general, experimental data are in good agreement with that model, considering the surface-to-volume ratio of the materials.^14^ Additionally, Bi_2_Te_3_ nanowires and 3D Bi_2_Te_3_ networks have shown plasmon resonances, whose position depends on the nanowire interactions and can be tuned.^16^ Although individual NWs or thin films of Bi_2_Te_3_ show reduced thermoelectric performance as a consequence of the highly conducting surface channel, ordered 3D NW networks of Bi_2_Te_3_ have shown competitive thermoelectric performance due to a moderately high Seebeck coefficient and a strongly reduced thermal conductivity.^15^ Heat transport was hampered in the transversal joints of the cross-linked tubular structure.^15^ So, these 3D NW networks provide a platform to develop geometries not achieved before that can be tuned at the nanoscale, allowing the fabrication of metamaterials in a controlled manner, showing different properties and improved characteristics compared with bulk or conventional 1D nanowire counterparts. In this work, we investigate the characteristics of electrical transport at low temperature in the spatial structure of the NW network with two orthogonal current paths.

## Discussion

### Morphology and Crystal Structure

The EDX analysis showed that the sample had a stoichiometric composition consisting of Bi_2.0_Te_3.0_ within a 5% error; see Figure S3a (Supporting Information). This was confirmed by Raman spectroscopy, Figure S3b, when adjusting the deposition parameters to obtain the desired stoichiometry ratio 2:3 of Bi:Te. TEM analysis (Figure S2a–c) and XRD diffraction (Figure S2d) confirmed that the Bi_2_Te_3_ NWs of the network present a preferred crystal orientation along [110].

**Figure 1:**
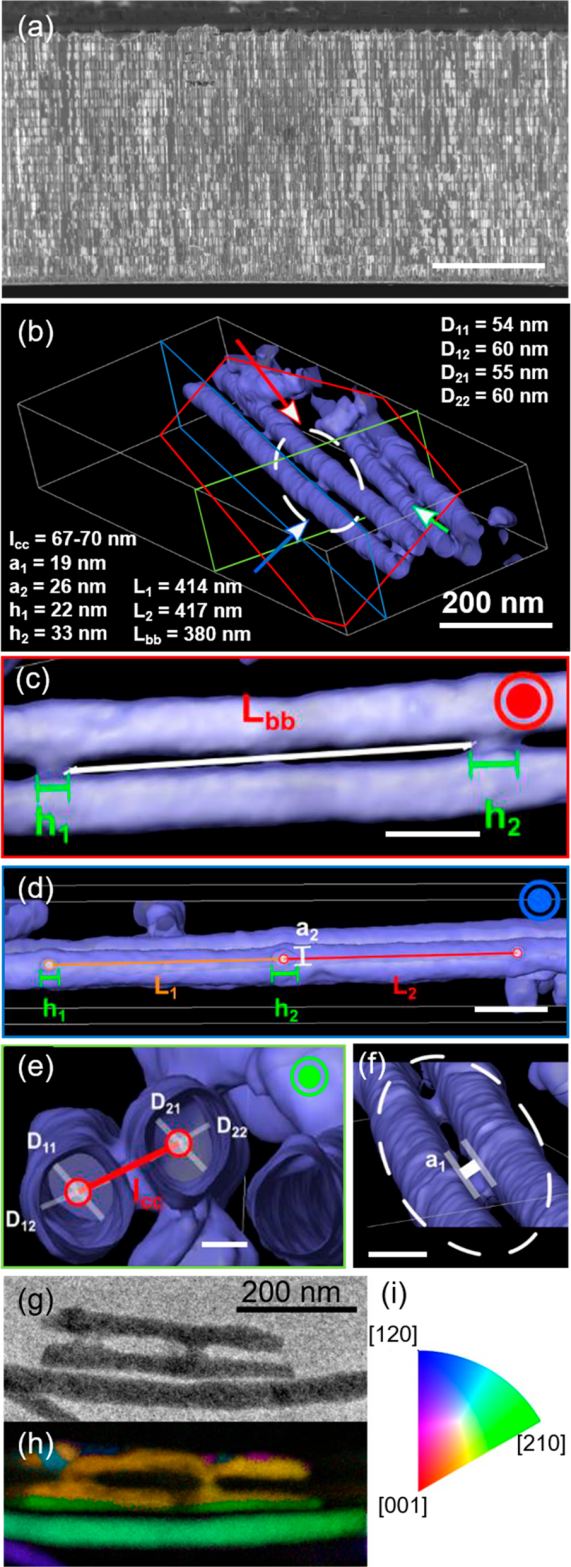
(a) Cross-view SEM image of the 3D Bi_2_Te_3_ nanonetwork still embedded in the alumina membrane (as measured); scale bar is 5 μm. (b) Volume rendering reconstruction of the free-standing 3D nanonetwork. (c–f) Reconstruction views: (c) longitudinal and (d) transversal slices through (b) with respect to the longitudinal nanowires. Scale bar: 100 and 50 nm, respectively. (e) View from the side indicated by the blue arrow in (b). Scale bar: 100 nm. (f) Zoom-in on the area marked with a circle in (b). Scale bar: 100 nm. (g) TEM image and (h) orientation map (found colored orientation + reliability + index map, 250 × 100 pixels) of one and two adjacent Bi_2_Te_3_ NWs. (i) Color map of the different symmetrically independent crystallographic directions. The crystallographic direction is displayed along the *z*-axis, the zone-axis of the diffraction pattern.

Figure 1a shows a SEM cross-section image with wide field of view of the free-standing 3D-Bi_2_Te_3_ nanonetwork of period 417 nm still embedded in the anodic aluminum oxide (AAO) membrane. No noticeable defects were observed in the NW nanonetworks. This is further supported with the detailed images of the empty and filled 3D AAO (Figure S1a and S1b) showing both good periodic structure of the 3D AAO and complete filling of 3D porous structure with Bi_2_Te_3_. This sample consisted of nanowires measuring 11.2 μm in length and around 55 nm in diameter. The transversal joints connecting the NWs were oval in shape, measuring around 30 nm in height and 20 nm in width. SEM images of the samples fabricated with period *L* = 269 and 580 nm are shown in Figure S1d and S1e, respectively. The total length of the nanowires, in each case, was 7.9 and 10.5 μm.

The connectivity of the free-standing 3D-Bi_2_Te_3_ nanonetwork was further determined with electron tomography (Figure 1). The reconstruction of a portion of the 3D-Bi_2_Te_3_ nanonetwork (Figure 1b) showed the continuous paths inside the volume of the nanowires and the joints building the transversal connections. Measurements of the different elements that form the network, including the longitudinal nanowires and the transversal connections, were done in different positions and from different perspectives (Figure 1c–f). Figure 1c shows a longitudinal slice of the nanowire through its volume, as indicated by the red plane in Figure 1b. In Figure 1c, the internal distance between transversal channels was *L*_bb_ = 380 nm, slightly less than the period. The height of two different connections, when measured along the nanowire axis, was *h*_1_ = 22 and *h*_2_ = 33 nm; thus, the average height of the joints was taken as 28 ± 5 nm. Figure 1d displays the reconstructed 3D nanonetwork from the perspective indicated by the blue arrow in Figure 1b. The period (taken from center to center of two transversal connections) was measured from the image in Figure 1d at two different positions of a nanowire (*L*_1_ and *L*_2_), resulting in 414 and 417 nm, respectively. Figure 1e shows a transversal slice at the position indicated by the green plane in Figure 1b, containing the transversal connections. From this perspective, the diameter of two different nanowires (*D*_1_ and *D*_2_ in Figure 1e) was measured at two different positions, D_11_–D_12_ and D_21_–D_22_, yielding an average diameter of 57 ± 5 nm. The lateral separation between the nanowire centers was *I*_cc_ = 67 ± 5 nm. Finally, Figure 1f shows an enlarged view of a transversal joint (white oval in Figure 1b). The average width of the bridges between the nanowires was 22 ± 5 nm (*a*_1_ = 19 and *a*_2_ = 26 nm, shown in Figure 1f and d, respectively). The tomography analysis corroborated the dimensions extracted from the SEM images and confirmed the homogeneity and cross-linking of the structure via the transversal joints.

Different orientation maps on different NWs were acquired across the TEM grids coated with lacey C as a support. An example is shown in Figure 1g and 1h where three different NWs can be observed. It is important to highlight that the same orientation is maintained through the junctions. Here, the [210] direction is found, which is equivalent to [120] for this crystal system; thus, [110] is along the longitudinal direction of the NW. Smaller grains are seen as well, which may be broken fragments of other NWs that may be attached due to the sonication of the sample before the TEM grid preparation.

### In-Plane Resistance and Magnetoresistance

**Figure 2:**
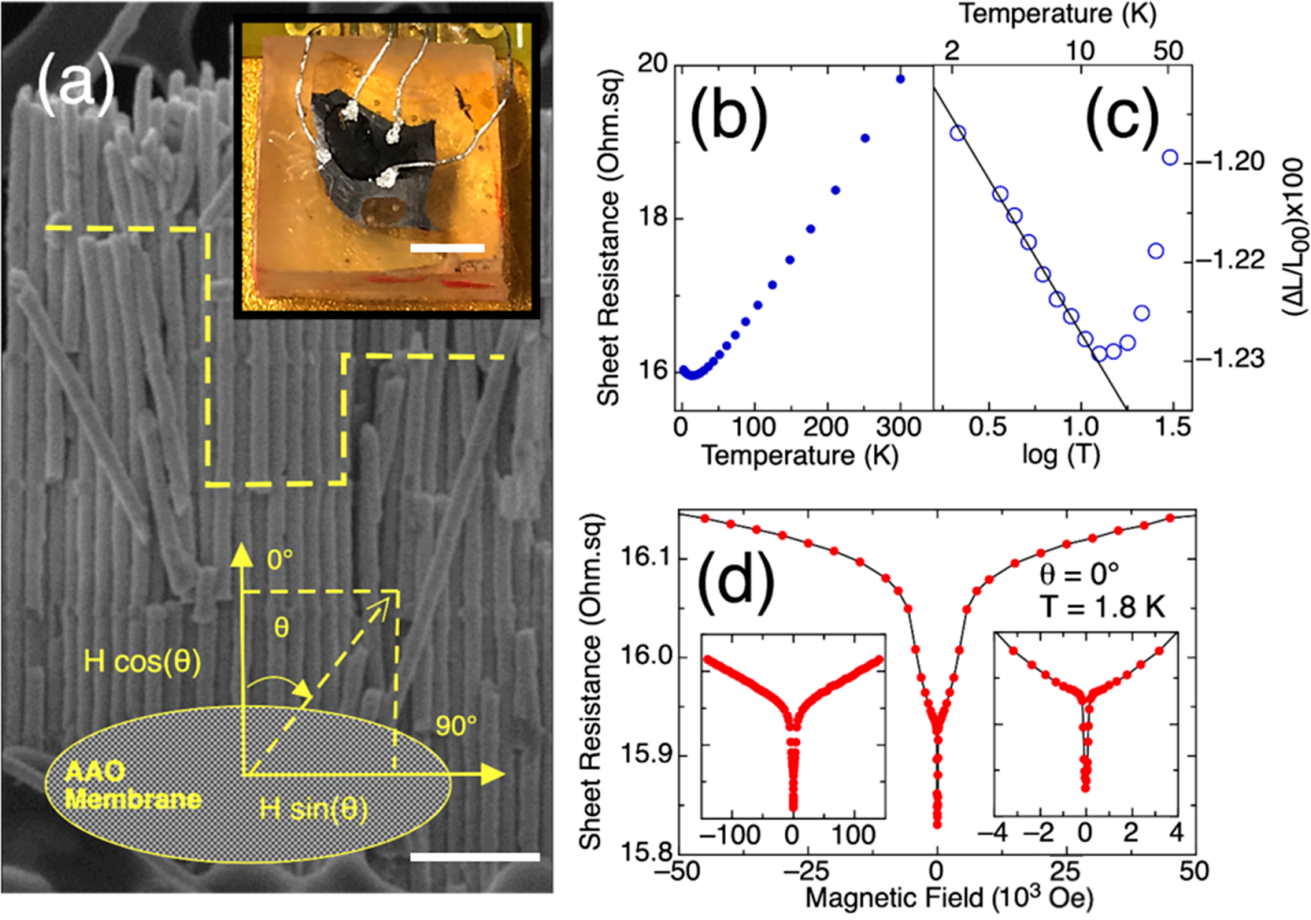
(a) Schematic representation of current paths and the relative orientation of the AAO and magnetic field superimposed on an SEM image of the sample. Scale bar = 500 nm. The inset corresponds to a photograph of the sample contacted for resistance measurements. Scale bar: 3 mm. (b) Temperature dependence of the resistance of the 3D-Bi_2_Te_3_ nanonetwork inside the 3D porous alumina (3D-AAO). (c) Plot of the difference conductance as a function of the temperature logarithm. The line shows the linear fit of the data below 10 K. *L*_00_ = *e*^2^/(2π^2^ℏ). (d) Magnetoresistance at 1.8 K and zero tilt. Left inset: the magnetoresistance in the full measured range from −150 000 to 150 000 Oe. Right inset: detail of the low-field sharp feature.

The temperature dependence of the resistance of the 3D-Bi_2_Te_3_ NW nanonetwork was measured by applying the electrical current in the direction parallel to the plane of the AAO membrane (Figure 2a). In this way, the electrical current needs to cross the transversal channels connecting the nanowires in the network, as indicated schematically in Figure 2a. The data in Figure 2b–d correspond to the sample with period 471 nm. The resistance decreased steadily as the temperature decreased from 300 K to about 10 K (Figure 2b). Below about 10 K the resistance increased again with the logarithm of the temperature. This resistance-to-temperature dependence is typically associated with a semiconducting regime. However, taking into account that our 3D-Bi_2_Te_3_ nanonetwork has a carrier density of around 10^20^ cm^–3^,^15^ we should refer to it as a degenerated semiconductor, and thus, another explanation for this behavior should be found.

Analyzing the difference conductance, Δ*L*, at the low-temperature range (Figure 2c), we observed the same linear tendency with the log(*T*) below 10 K. This behavior is compatible with a localization situation, which is not usually discussed in the literature for bismuth telluride. The difference conductance can be described in that case by an inelastic-scattering relaxation time of the form τ_*i*_ ∼ *T*^–*p*^, where *p* = 0(1).^27,28^ In this way, the slope of the Δ*L* “vs” log(*T*) curve can be written as *N*[*e*^2^/2π^2^ℏ]*αp*, where α may adopt different values depending on the interaction mechanism for electrons and dimensionality.^27,29^ An integer factor *N* has been added to account for the number of parallel transversal current paths realized in the 3D-Bi_2_Te_3_ NW nanonetwork. Estimating *N* = 27 from the morphological characteristics of the sample, we reach *αp* = 1.8 with a 20% uncertainty. This value is in very good agreement with the ones observed in thin metallic films showing 2D localization.^29^ The consistency of the obtained temperature exponent was confirmed by measuring samples having different periods. Samples with a period of 417 (data of Figure 2), 269, and 580 nm were investigated, resulting in values of *αp* 1.8, 2.0, and 2.2, respectively. The values of Δ*L* per transversal channel and SEM images of the corresponding samples are shown in the Supporting Information (Figure S1f). The realization of Anderson’s localization in the nanostructured 3D nanowire network suggests that additional localized energy states close to the Fermi level are created as a consequence of the thin connections between the NWs, thus affecting or modifying the energy level structure of the bismuth telluride material. The particular spatial arrangement of the NWs results in the localization phenomena observed in the resistance measurements presented in this work. Moreover, it correlates with the increase of the Seebeck coefficient previously reported in the 3D-Bi_2_Te_3_ NW nanonetworks.^15^ The values of the Seebeck coefficient measured for these NW nanonetworks are 18–20% larger than those obtained in isolated NWs.^8^ Measurements performed on 3D nanonetworks of different period between transversal channels, *L*, showed in-plane Seebeck coefficient values at room temperature ranging from −110 to −145 μV/K, their magnitude increasing with increasing *L*. The lowest Seebeck coefficient of −110 ± 10 μV/K corresponds to a 3D-Bi_2_Te_3_ NW nanonetwork with *L* ∼ 200 nm, whereas, for *L* ∼ 700 nm, we report a value of −145 ± 15 μV.

**Figure 3:**
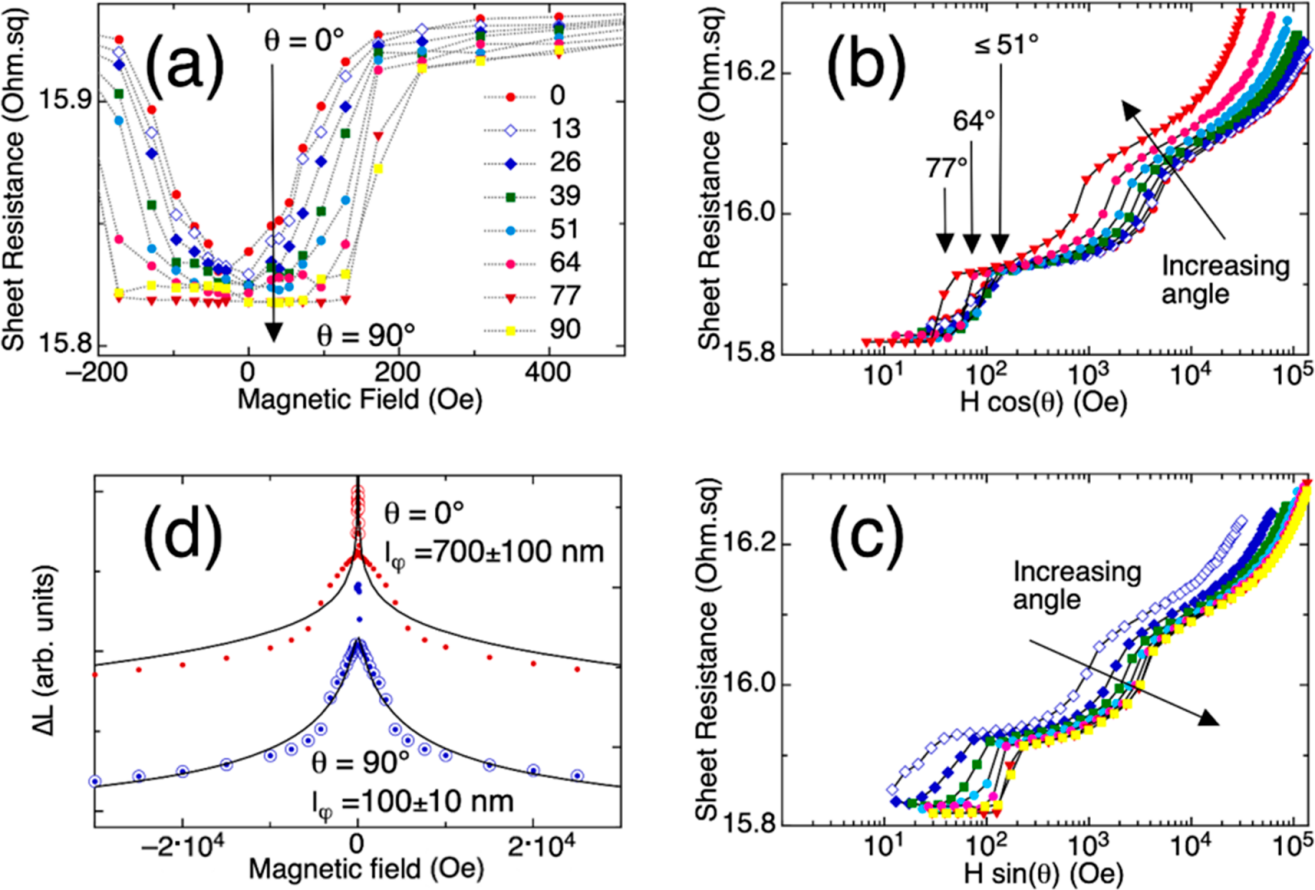
(a) Low-field feature measured at several tilt angles. (b) Magnetoresistance curves versus the component of the field parallel to the length of one NW. (c) Magnetoresistance curves versus the component of the field transversal to the length of the NWs. (d) Fitting of the magnetoresistance data to the HLN model. Open circles indicate the data points used for fitting.

Magnetoresistance was measured at 1.8 K. A very sharp downward peak is observed near zero field (Figure 2d) arising from the weak antilocalization (WAL) effect. The WAL effect represents destructive interference between wave functions of electrons propagating in opposite directions^30,31^ and can arise from bulk states in materials having a strong spin–orbit coupling, which causes the spins to rotate in opposite directions, and from the surface states of topological insulators, with opposite spin-momentum locking. As the external magnetic field is increased, the WAL effect is destroyed due to the alignment of the spins with the applied field, and the classical regime is recovered, with the resistance increasing with the increase of the applied field. Unlike the WAL effect reported in other Bi_2_Te_3_ single NW or thin film samples, the increase in resistance with magnetic field takes place in two steps (Figure 2d). At magnetic field values of 25 000 Oe and above, the magnetoresistance measured in the 3D NW nanonetwork is almost linear (Figure 2d left inset). The feature close to zero field is very sharp, about 200 Oe wide (Figure 2d right inset). However, the peak changes from a sharp increase to a much softer trend at intermediate magnetic fields, which was not reported previously. But, furthermore, the two steps in the magnetoresistance curve (occurring at low/intermediate and intermediate/high magnetic fields, respectively) are affected by the tilt angle in a different manner. Figure 3 shows the measured sheet resistance at different tilt angles of the sample of a 417 nm period inside the magnetic field, from the direction perpendicular to the sample plane (0°) to the direction parallel to the surface of the AAO membrane (90°). The relative orientation of the sample and magnetic field is schematically shown in Figure 2a. The feature closest to zero field collapses to a flat minimum resistance as the tilt angle changes from 0° to 90° (Figure 3a). The WAL effect associated with two-dimensional (e.g., surface) transport is sensitive only to the component of the magnetic field normal to the direction of the current. In previous reports, it has been shown how the WAL peak disappears gradually as the current direction becomes parallel to the magnetic field.^32^ However, it is worth noting that when rotating the sample to orient the plane of the sample with the field, in a configuration where both the electrical current and the magnetic field are parallel, the nanowires of the 3D network are now the ones perpendicular to the magnetic field, which should be considered as an additional transport channel contributing to the WAL effect even in a 90° angle configuration. Consequently, the effect of the tilt angle on both magnetoresistance steps is further emphasized in Figure 3b and c where the dependence with the field components normal to the AAO surface, *H* cos(θ), and parallel to the AAO surface, *H* sin(θ), is presented. For moderate tilt angles up to 64°, the magnetoresistance curves collapse on each other, meaning the resistance behavior is the same in the low-field region (*H* below about 200 Oe in Figure 3a) when represented against *H* cos(θ) (Figure 3b). This indicates a stronger contribution to the WAL effect of current flowing in the plane of the AAO membrane, across the transversal junctions between NWs. For tilt angles higher than about 64°, the low-field magnetoresistance does not collapse anymore with *H* cos(θ), thus suggesting a contribution of current flowing both along and across the nanowires. At intermediate and high magnetic fields, above about 200 Oe in Figure 3a, the magnetoresistance curves remain closer to each other, though not completely collapsing, when represented against *H* sin(θ) (Figure 3c). This could indicate a conductive component along the individual nanowires. These results suggest that there are two mutually perpendicular conductive channels in the 3D NW networks. For low *H*, the current preferably flows along the plane of the AAO, where transport could be associated with topological surface states, giving rise to the marked WAL signature^33,34^ in Figure 3b. At intermediate and high fields, a current component along the individual nanowires can be associated with a less intense WAL effect. In this case, a component arising due to the spin–orbit interaction of bulk states^35,36^ could be relevant. In Figure S1, both perpendicular transport orientations inside the ordered 3D NW nanonetwork are depicted.

To gain further insight into the observed phenomena, we have fitted the 0° and 90° curves of the magnetoconductance employing the Hikami–Larkin–Nagaoka (HLN) model,^37^Figure 3d. The values of the coherence length, *l*_φ_, obtained were 700 ± 100 nm for the low-field peak at 0° tilt and 100 ± 10 nm for the intermediate feature at 90° tilt. This value of *l*_φ_, for 0° tilt, would correspond to coherence across approximately 10 nanowire junctions. However, for the 90° curve, we found the best fit at the intermediate fields (Figure 3d). The sharpening of the intermediate step at 90° tilt would further reinforce the idea of transport taking place along the NWs. The value obtained for *l*_φ_, in this case, around 100 nm, is significantly smaller than the distance between transverse joints along the nanowires, which is almost 4 times bigger, making clear that there is no interaction between the different transversal planes in this direction. Moreover, this also points out the participation of bulk states along the nanowires. More information about the transport through clean bulk bands would be required to fit the magnetoresistance over the entire field range. The detailed impact of several possible scattering mechanisms in such a confined structure would be needed. Such models have not yet been fully developed and will not be addressed in this work.

## Conclusions

The resistance and magnetoresistance of the 3D-Bi_2_Te_3_NW nanonetworks have been investigated at low temperatures. The interconnectivity of the NWs was demonstrated with detailed electron tomography. At low temperatures, the resistance increases logarithmically with a decrease in temperature, in agreement with the Anderson model for localization. The temperature exponent of the resistance was measured in samples with distances between transversal planes of 417, 269, and 580 nm, resulting in values of 1.8, 2.0, and 2.2, respectively. These values are consistent with localized transport in 2D, realized in individual parallel channels across the NW nanonetwork. The magnetoresistance data at 1.8 K showed a double WAL feature that we could associate with transport both across transversal channels and along individual NWs. The coherence length in the transversal direction is approximately 700 nm, which corresponds to 10 transversal NW junctions. In the direction along the NWs the coherence length is approximately 100 nm, roughly one-fourth of the distance separating junctions in a single NW. The realization of a confinement effect because of the tight transversal joints could be related to the enhancement of the Seebeck coefficient in the 3D-Bi_2_Te_3_ NW nanonetwork.

## Experimental Section

3D NW structures were fabricated via template-assisted electrochemical deposition. The templates used were three-dimensionally interconnected anodic aluminum oxide (3D-AAO) produced by the technique described by Martín et al.^17−19^ Briefly, it consists of a two-step anodization process in sulfuric acid, the first of which defines the ordering of the nanopores along the surface of the aluminum oxide in a highly ordered hexagonal array, and the second produces the nanopores with pulsed voltage anodization. In the second step, the voltage is accurately controlled to alternate between conditions that produce mild or hard anodization along the length of the pores. After removal of the remaining aluminum and barrier layer, the AAO is etched in phosphoric acid, with mild and hard anodized regions having different etch rates. 3D-AAO structures are produced carefully controlling the anodization parameters and the etching process. Nanochannels connecting the nanopores are formed as a consequence of the hard anodization step of the AAO, and they all appear in planes transversal to the length of the nanopores at regular intervals. The distance between consecutive planes of connecting nanochannels, also termed the period, *L*, can be finely tuned by changing the pulses of the second anodization step. Each nanopore is connected to its six closest neighbors with transversal nanochannels. In this work, three different structures were fabricated with period *L* = 580, 417, and 269 on average with ±10 nm uncertainty. Periodic pulses of 540, 360, and 180 s were, respectively, applied under mild anodization conditions, and 2 s under hard anodization conditions.

Bi_2_Te_3_ was grown by pulsed electrodeposition inside the 3D-AAO structure in a three-electrode cell configuration as reported in refs (20 and 21). The deposition process was controlled with a potentiostat (Autolab PGSTAT 302N) with Nova 2.0 software. The deposition bath consisted of 9 mM Bi^3+^ (bismuth pieces, 99.999% Sigma-Aldrich), 10 mM HTeO^2+^ (tellurium powder, 99.999% Sigma-Aldrich), and 1 M HNO_3_ (65%, Panreac). The net applied voltage, against the Ag/AgCl reference electrode, was 18 mV, in the case of the 3D networks with *L* = 269 and 417 nm, and 41 mV, for the 3D network with *L* = 580 nm. A more detailed description of the process is provided in the Supporting Information. After deposition, the Bi_2_Te_3_-filled 3D-AAO template was detached from the holder by immersion in acetone. Free-standing 3D-Bi_2_Te_3_ structures were obtained by selectively dissolving the AAO in a mixture of 7 wt % H_3_PO_4_ (85 wt %, Sigma-Aldrich) and 1.8 wt % CrO_3_ (99.99%, Sigma-Aldrich) for 24 h.

Morphological characterization was carried out with high-resolution scanning electron microscopy (HR-SEM, FEI Verios 460). The chemical composition was determined by energy dispersive X-ray spectrometry (EDX, Hitachi S-300N), assuming an error of 5% in the atomic percentage. Crystal structure and orientation were determined with an X-ray diffractometer (XRD, Philips X’Pert PANalytical with Cu Kα radiation, 0.15418 nm). Electron tomography experiments were conducted in a Titan Themis transmission electron microscope (TEM) at 200 keV, retrieving the reconstructions from 15 to 30 projections through a total variation (TV) minimization algorithm run in MATLAB. The alignment of the projections before the reconstruction was carried out in the Thermo Fisher Inspect 3D software and the TomoJ plugin of the ImageJ software. The reconstruction and visualization of the volumes were performed using Thermo Fisher Avizo software. An orientation map of the crystalline structure along the NWs was obtained by using 4D-STEM. The electron diffraction maps were acquired in a JEOL 2100 TEM with an LaB_6_ cathode operated at 200 kV. As the electron probe scans a selected area, a diffraction pattern in each pixel of scanned region I was saved. In this way, the collected electron diffraction patterns can be compared to simulated ones and identify the crystallographic orientation that fits best, an algorithm called template-matching. The figure of merit to distinguish between the different orientation possibilities, also known as index and reliability factors, can be found elsewhere.^22,23^ TEM mode, spot 5, alpha 3, and a 10 μm condenser aperture were selected to configure a small (5–6 nm) and low convergence angle (1 mrad) electron beam. The diffraction maps were acquired by means of the ASTAR system provided by NanoMEGAS SPRL using an optical CCD camera placed at the binocular position. A precession angle of 0.65° was applied to the electron probe to obtain uniform intensities, related to the structure factor, inside the reflection disks and to increase the number of reflections available in each pattern. This enables an improvement of the results of the template-matching algorithm implemented in the ASTAR system.^24,25^ The crystallographic information file of Bi_2_Te_3_ from Feutelais et al.^26^ was used for the simulation of the diffraction patterns (space group *R*3̅*m* with *a* = 4.395(3) Å and *c* = 30.44(1) Å).

Angle-dependent magnetoresistance measurements were performed in a DynaCool cryostat using the electrical transport option, rotator probe, and automated switchbox ASB102 (all from Quantum Design). The measurement was performed in the Van der Pauw geometry with the 3D-Bi_2_Te_3_ nanonetwork inside the AAO template and embedded in epoxy resin to provide mechanical stability at cryogenic temperatures. Once embedded in resin, the top surface of the AAO was delicately polished to ensure that the NWs were clean and electrically accessible. Electrical contacts were made on the top of the AAO with freshly cleaned indium wire (Ø = 0.15 mm, 99%, HMW Hauner GmbH & Co. KG) pressed onto it. The spacing distance between contacts ranges from approximately 1.5 to 2 mm for the different samples measured in this work. The ohmic character of the contacts was assessed with the phase shift between the AC excitation current (of frequency between 15 and 60 Hz depending on the sample) and the measured AC voltage. It was in all cases between 0.1° and 0.01°, evidencing excellent ohmic character of the contacts (for more details, see the Supporting Information).

The in-plane Seebeck coefficient was obtained at room temperature in a custom-made measurement system, where an in-plane thermal gradient can be established. Measurements were performed upon embedding the samples in resin.

## Supporting Information Available

Additional information material about the fabrication process and characterization of the 3D NW networks: detailed description of the fabrication steps and sample preparation, SEM images of the 3D NW networks with different periods, *L*, and their corresponding conductance measurements, TEM and XRD results about the crystal structure of the 3D-Bi_2_Te_3_ NW nanonetworks, and EDAX and Raman analysis, to study their chemical composition (PDF)

## ABBREVIATIONS

NWs: nanowires
TIs: topological insulators
AAO: anodic aluminum oxide (also known as nanohole alumina arrays, −NAA–, or nanoporous anodized alumina platforms, −NAAP−)
3D-AAO: three-dimensional interconnected nanopores in anodic alumina
TV: total variation
*D*: nanowire diameter
*L*: distance from center to center of two transversal connections
*L*_bb_: the distance between connections excluding the transversal channels
*h*: height of transversal connections
*a*: width of the transversal connectors
*I*_cc_: distance between the center of the nanowires
Δ*L*: difference conductance
*N*: number of parallel transversal current paths
WAL: weak antilocalization
*H*: applied magnetic field
Oe: Oersted
HLN: Hikami–Larkin–Nagaoka

## References

[ref1] Chen R.; Lee J.; Lee W.; Li D. Thermoelectrics of Nanowires. Chem. Rev. 2019, 119, 9260–9302. 10.1021/acs.chemrev.8b00627. 30882214

[ref2] Domínguez-Adame F.; Martín-González M.; Sánchez D.; Cantarero A. Nanowires: A Route to Efficient Thermoelectric Devices. Physica E: Low-dimensional Systems and Nanostructures 2019, 113, 213–225. 10.1016/j.physe.2019.03.021.

[ref3] Nielsch K.; Bachmann J.; Kimling J.; Böttner H. Thermoelectric Nanostructures: from Physical Model Systems Towards Nanograined Composites. Adv. Energy Mater. 2011, 1, 713–731. 10.1002/aenm.201100207.

[ref4] Caballero-Calero O.; Martín-González M. Thermoelectric Nanowires: A Brief Prospective. Scripta Materialia 2016, 111, 54–57. 10.1016/j.scriptamat.2015.04.020.

[ref5] Rojo M. M.; Abad B.; Manzano C. V.; Torres P.; Cartoixà X.; Alvarez F. X.; Gonzalez M. M. Thermal Conductivity of Bi 2 Te 3 Nanowires: How Size Affects Phonon Scattering. Nanoscale. 2017, 9, 6741–6747. 10.1039/C7NR02173A. 28485423

[ref6] Liu C. W.; Wang Z.; Qiu R. L.; Gao X. P. Development of Topological Insulator and Topological Crystalline Insulator Nanostructures. Nanotechnology. 2020, 31, 192001 10.1088/1361-6528/ab6dfc. 31962300

[ref7] Kojda D.; Mitdank R.; Weidemann S.; Mogilatenko A.; Wang Z.; Ruhhammer J.; Kroener M.; Töllner W.; Woias P.; Nielsch K.; Fischer S. F. Surface Effects on Thermoelectric Properties of Metallic and Semiconducting Nanowires. Physica Status Solidi (A) 2016, 213, 557–570. 10.1002/pssa.201532464.

[ref8] Shin H. S.; Hamdou B.; Reith H.; Osterhage H.; Gooth J.; Damm C.; Rellinghaus B.; Pippel E.; Nielsch K. The Surface-to-Volume Ratio: A Key Parameter in the Thermoelectric Transport of Topological Insulator Bi 2 Se 3 Nanowires. Nanoscale. 2016, 8, 13552–13557. 10.1039/C6NR01716A. 27362294

[ref9] Bäßler S.; Hamdou B.; Sergelius P.; Michel A. K.; Zierold R.; Reith H.; Gooth J.; Nielsch K. One-Dimensional Edge Transport on the Surface of Cylindrical BixTe3–ySey Nanowires in Transverse Magnetic Fields. Appl. Phys. Lett. 2015, 107, 181602 10.1063/1.4935244.

[ref10] Nolas G. S.; Sharp J.; Goldsmid J. Thermoelectrics: Basic Principles and New Materials Developments; Springer Science & Business Media, 2001, 10.1007/978-3-662-04569-5.

[ref11] Zhang H.; Liu C. X.; Qi X. L.; Dai X.; Fang Z.; Zhang S. C. Topological Insulators in Bi2Se3, Bi2Te3 and Sb2Te3 with a Single Dirac Cone on the Surface. Nature Physics. 2009, 5, 438–442. 10.1038/nphys1270.

[ref12] Chen Y. L.; Analytis J. G.; Chu J. H.; Liu Z. K.; Mo S. K.; Qi X. L.; Zhang H. J.; Lu D. H.; Dai X.; Fang Z.; Zhang S. C.; Fisher I. R.; Hussain Z.; Shen Z. X. Experimental Realization of a Three-Dimensional Topological Insulator, Bi2Te3. Science. 2009, 325, 178–181. 10.1126/science.1173034. 19520912

[ref13] Li Y. Y.; Wang G.; Zhu X. G.; Liu M. H.; Ye C.; Chen X.; Wang Y. Y.; He K.; Wang L. L.; Ma X. C.; Zhang H. J.; Dai X.; Fang Z.; Xie X. C.; Liu Y.; Qi X. L.; Jia J. F.; Zhang S. C.; Xue Q. K. Intrinsic Topological Insulator Bi2Te3 Thin Films on Si and Their Thickness Limit. Adv. Mater. 2010, 22, 4002–4007. 10.1002/adma.201000368. 20648518

[ref14] Hinsche N. F.; Zastrow S.; Gooth J.; Pudewill L.; Zierold R.; Rittweger F.; Rauch T.; Henk J.; Nielsch K.; Mertig I. Impact of the Topological Surface State on the Thermoelectric Transport in Sb2Te3 Thin Films. ACS Nano 2015, 9, 4406–4411. 10.1021/acsnano.5b00896. 25826737

[ref15] Ruiz-Clavijo A.; Caballero-Calero O.; Manzano C. V.; Maeder X.; Beardo A.; Cartoixà X.; Alvarez F. X.; Martín-González M. 3D Bi2Te3 Interconnected Nanowire Networks to Increase Thermoelectric Efficiency. ACS Applied Energy Materials 2021, 4, 13556–13566. 10.1021/acsaem.1c02129. 35647490 PMC9127787

[ref16] Caballero-Calero O.; Ruiz-Clavijo A.; Manzano C. V.; Martín-González M.; Armelles G. Plasmon Resonances in 1D Nanowire Arrays and 3D Nanowire Networks of Topological Insulators and Metals. Nanomaterials. 2023, 13, 154 10.3390/nano13010154. PMC9823705 36616063

[ref17] Martín J.; Martín-González M.; Francisco Fernandez J.; Caballero-Calero O. Ordered Three-Dimensional Interconnected Nanoarchitectures in Anodic Porous Alumina. Nat. Commun. 2014, 5, 5130 10.1038/ncomms6130. 25342247 PMC4770565

[ref18] Ruiz-Clavijo A.; Caballero-Calero O.; Martín-González M. Revisiting Anodic Alumina Templates: From Fabrication to Applications. Nanoscale. 2021, 13, 2227–2265. 10.1039/D0NR07582E. 33480949

[ref19] Manzano C. V.; Rodríguez-Acevedo J.; Caballero-Calero O.; Martín-González M. Interconnected Three-Dimensional Anodized Aluminum Oxide (3D-AAO) Metamaterials Using Different Waveforms and Metal Layers for RGB Display Technology Applications. Journal of Materials Chemistry C 2022, 10, 1787–1797. 10.1039/D1TC05209H.

[ref20] Ruiz-Clavijo A.; Caballero-Calero O.; Martín-González M. Three-Dimensional Bi2Te3 Networks of Interconnected Nanowires: Synthesis and Optimization. Nanomaterials. 2018, 8, 345 10.3390/nano8050345. 29783697 PMC5977359

[ref21] Manzano C. V.; Polyakov M. N.; Maiz J.; Aguirre M. H.; Maeder X.; Martín-González M. Pulsed Current-Voltage Electrodeposition of Stoichiometric Bi2Te3 Nanowires and Their Crystallographic Characterization by Transmission Electron Backscatter Diffraction. Sci. Technol. Adv. Mater. 2019, 20, 1022–1030. 10.1080/14686996.2019.1671778. 31723369 PMC6830284

[ref22] Rauch E. F.; Dupuy L. Rapid Spot Diffraction Patterns Idendification through Template Matching. Archives of Metallurgy and Materials 2005, 50, 87–99.

[ref23] Eggeman A. S.; Krakow R.; Midgley P. A. Scanning Precession Electron Tomography for Three-Dimensional Nanoscale Orientation Imaging and Crystallographic Analysis. Nat. Commun. 2015, 6 (1), 7267 10.1038/ncomms8267. 26028514 PMC4458861

[ref24] Portillo J.; Rauch E. F.; Nicolopoulos S.; Gemmi M.; Bultreys D. Precession Electron Diffraction Assisted Orientation Mapping in the Transmission Electron Microscope. Mater. Sci. Forum 2010, 644, 1–7. 10.4028/www.scientific.net/MSF.644.1.

[ref25] Viladot D.; Véron M.; Gemmi M.; Peiró F.; Portillo J.; Estradé S.; Mendoza J.; Llorca-Isern N.; Nicolopoulos S. Orientation and Phase Mapping in the Transmission Electron Microscope Using Precession-Assisted Diffraction Spot Recognition: State-of-the-Art Results. J. Microsc. 2013, 252, 23–34. 10.1111/jmi.12065. 23889078

[ref26] Feutelais Y.; Legendre B.; Rodier N.; Agafonov V. A Study of the Phases in the Bismuth - Tellurium System. Mater. Res. Bull. 1993, 28, 591–596. 10.1016/0025-5408(93)90055-I.

[ref27] Anderson P.; Abrahams E.; Ramakrishnan T. Possible Explanation of Nonlinear Conductivity in Thin-Film Metal Wires. Phys. Rev. Lett. 1979, 43, 718 10.1103/PhysRevLett.43.718.

[ref28] Bergmann G. Weak Localization in Thin Films: A Time-of-Flight Experiment with Conduction Electrons. Phys. Rep. 1984, 107, 1–58. 10.1016/0370-1573(84)90103-0.

[ref29] Van Haesendonck C.; Bruynseraede Y.; Deutscher G. Two-Dimensional Localization in Thin Copper Films. Phys. Rev. Lett. 1981, 46, 565 10.1103/PhysRevLett.46.565.

[ref30] Gracia-Abad R.; Sangiao S.; Bigi C.; Kumar Chaluvadi S.; Orgiani P.; De Teresa J. M. Omnipresence of Weak Antilocalization (WAL) in Bi2Se3 Thin Films: A Review on Its Origin. Nanomaterials. 2021, 11, 1077 10.3390/nano11051077. 33922019 PMC8143463

[ref31] Khalaf E.; Ostrovsky P. Boundary Scattering Effects on Magnetotransport of Narrow Metallic Wires and Films. Phys. Rev. B 2016, 94, 165431 10.1103/PhysRevB.94.165431.

[ref32] Shrestha K.; Shrestha K.; Graf D.; Marinova V.; Lorenz B.; Chu C. W. Weak Antilocalization Effect Due to Topological Surface States in Bi2Se2. 1Te0. 9. J. Appl. Phys. 2017, 122, 145901 10.1063/1.4997947.

[ref33] Krieg J.; Giraud R.; Funke H.; Dufouleur J.; Escoffier W.; Trautmann C.; Toimil-Molares M. E. Magnetotransport Measurements on Bi2Te3 Nanowires Electrodeposited in Etched Ion-Track Membranes. J. Phys. Chem. Solids 2019, 128, 360–366. 10.1016/j.jpcs.2018.02.002.

[ref34] Kim H. S.; Shin H. S.; Lee J. S.; Ahn C. W.; Song J. Y.; Doh Y. J. Quantum Electrical Transport Properties of Topological Insulator Bi2Te3 Nanowires. Curr. Appl. Phys. 2016, 16, 51–56. 10.1016/j.cap.2015.10.011.

[ref35] Sangiao S.; Marcano N.; Fan J.; Morellón L.; Ibarra M. R.; De Teresa J. M. Quantitative Analysis of the Weak Anti-Localization Effect in Ultrathin Bismuth Films. EPL (Europhysics Letters) 2011, 95, 37002 10.1209/0295-5075/95/37002.

[ref36] He H. T.; Wang G.; Zhang T.; Sou I. K.; Wong G. K.; Wang J. N.; Lu H. Z.; Shen S. Q.; Zhang F. C. Impurity Effect on Weak Antilocalization in the Topological Insulator Bi 2 Te 3. Physical review letters 2011, 106, 166805 10.1103/PhysRevLett.106.166805. 21599398

[ref37] Hikami S.; Larkin A. I.; Nagaoka Y. Spin-Orbit Interaction and Magnetoresistance in the Two-Dimensional Random System. Prog. Theor. Phys. 1980, 63, 707–710. 10.1143/PTP.63.707.

