# Supplementary material for: Localization and Directionality of Surface Transport in Bi2Te3 Ordered 3D Nanonetworks
Source: ACS Nano. 2023 Jul 6;17(17):16960–7. doi: 10.1021/acsnano.3c04160 (PMC10510701; doi:10.1021/acsnano.3c04160)
Supplement: Supplementary file 1 — nn3c04160_si_001.pdf [file nn3c04160_si_001.pdf]

Supplementary information for

Localization and directionality of surface transport in Bi2Te3 ordered 3D nano-networks.

Alejandra Ruiz-Clavijo§, Nicolás Pérez†,*, Olga Caballero-Calero§, Javier Blanco||,¥, Francesca Peiró||,¥, Sergi Plana-Ruiz||,¢, Miguel López-Haro‡, Kornelius Nielsch†, Marisol Martín-González§,*.

§ Instituto de Micro y Nanotecnología, IMN-CNM, CSIC (CEI UAM+CSIC) Isaac Newton 8, E-28760, Tres Cantos, Madrid, Spain

† Institute for Metallic Materials, IFW-Dresden, Helmholtzstrasse 20, 01069 Dresden, Germany

|| LENS-MIND, Department of Electronics and Biomedical Engineering, Universitat de Barcelona, 08028 Barcelona

¥ Institute of Nanoscience and Nanotechnology (IN2UB), Universitat de Barcelona, 08028 Barcelona, Spain

¢ Scientific & Technical Resources, Universitat Rovira i Virgili, 43007 Tarragona, Spain

‡ Departamento de Ciencia de los Materiales e Ingeniería Metalúrgica y Química Inorgánica, Facultad de Ciencias, Univer-sidad de Cádiz, Cádiz 11510, Spain

KEYWORDS: Metamaterials, bismuth telluride, 3D-AAO, Nano-network, surface transport, localization effects.

Fabrication procedure: 3D Bi_2Te_3 nano-networks

For the electrochemical deposition of Bi_2Te_3 inside the 3D-AAO structure, a layer of chromium (5 nm) and a layer of gold (150 nm) were evaporated on one side of the 3D-AAO. The gold layer was glued with silver paint to a copper holder and covered with varnish to define the working electrode of the electrochemical cell. The deposition was performed in a three-electrode electrochemical deposition chamber, with an Ag/AgCl (saturated KCl) reference electrode and Pt mesh as the counter electrode. The deposition bath consisted of 9 mM Bi^{3+}, 10 mM $HTeO^{2+}$ and 1 M HNO_3.

Then, Bi_2Te_3 was grown by pulsed electrodeposition, switching between potenciostatic and galvanostatic mode. The galvanostatic pulse was applied at zero current density for 0.1 s (time *off*). During this zero current density time, the system is left to rest, after ions are deposited and removed from the solution at the WE-electrolyte interface, and recovered, as the chemical species at the electrode interface are replaced by new ones that diffuse or migrate from other parts of the solution. Then, the process is turned back on for the application of the potetionstatic pulses for 1 s. Introducing a relaxation time during the electrodeposition process allows a more homogeneous growth front and good filling of the 3D porous structure of the AAO, as confirmed by the SEM images of Figure S1 a and c.

The applied potential versus Ag/AgCl in the potentiostatic part of the pulses was determined by a cyclic voltammetric (CV) curve performed prior to each deposition. The deposition voltage was chosen to be 60 mV higher than the reduction peak measured in the cyclic voltammogram before each deposition. The net applied voltage was 18 mV, against the Ag/AgCl reference electrode, in the case of the 3D networks with L = 269 and 417 nm and 41 mV, against the Ag/AgCl reference electrode, for the 3D network with L = 580 nm. Variations of the net applied deposition voltage are direct consequence of small displacements of the reduction peak recorded in the corresponding CVs.

Once the deposit was performed, the 3D–AAO matrix was detached from the copper holder by immersion in acetone. The free-standing 3D Bi_2Te_3 nanowire networks, shown in Fig 1S d-f, were obtained after the 3D–AAO matrix was dissolved in 7 wt % H_3PO_4 and 1.8 wt % H2CrO4 for 24 h.

Sample preparation: contacting the samples

Magnetoresistance measurements were carried at 1.8 K, to avoid cracking of the samples when subjected to such low temperatures, due to difference thermal expansion between the alumina membranes and Bi_2Te_3, the samples were further embedded in epoxy resin, as described in the main text. Then, the surface was polished to expose the nanowires and contact the sample. The sample was electrically contacted employing 99% pure Indium wire with a diameter of 0.150 mm. Indium is a highly ductile metal, therefore, the wires were attached to the surface sample at room temperature by applying pressure (as described in the main text). Another reason for choosing indium wire was to avoid any reaction between our bismuth telluride nanowires and the contacting metal (Au, Ag). Oxidation of the contacts is also avoided. The contacts remained well attached to the sample surface during the whole experiment until date, no further steps were required (such as annealing) to ensure good electrical contact. The spacing distance between contacts ranges from approximately 1.5 to 2 mm.

Dielectric barriers (and / or Schottky barriers for lower doping levels) may be an issue when contacting these materials, particularly in nanoflake or nanowire form. In our instrument we detect the phase shift between the AC excitation current (frequency between 15-60 Hz, depending on the case) and the measured voltage. In all cases that deviation was of the order of 10^{-1} to 10^{-2} degrees, which indicates excellent ohmic character. Additionally, we estimated the contact resistance by comparing 4-point and 2-point measurements. The overall size of the samples makes it difficult to use the transfer length method. It resulted in an estimated contact resistance ranging between 40 Ohm to 80 Ohm per contact. Having discarded the effect of dielectric barriers, we attribute the relatively high value to the very small effective contact area between the indium wire, which has an imprint of ca 0.2 mm^2, and the underlying very narrow nanowires.

Characterization of the 3D Bi_2Te_3 nano-networks

SEM

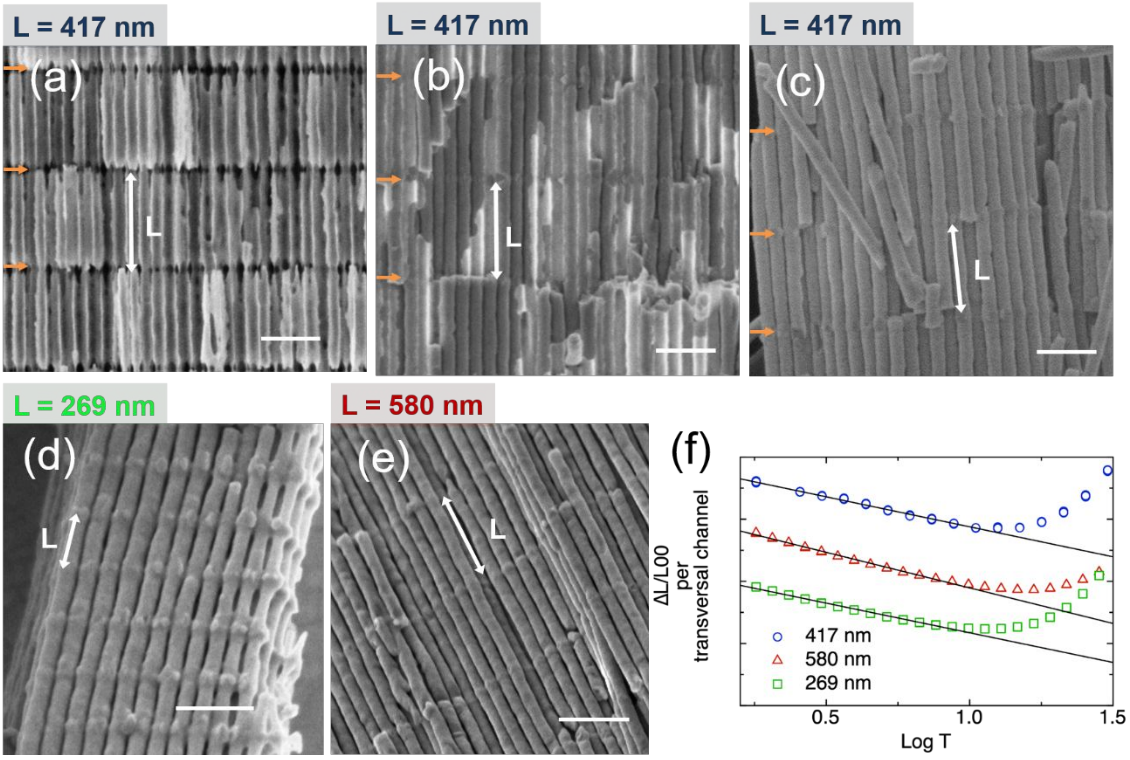

Figure S1. a) Cross-view SEM image of the 3D AAO template, transversal nano pore channels are indicated with orange arrows. b) Cross-view SEM image of the 3D Bi2Te3 nano-network embedded in the 3D AAO template. c) Free-standing 3D Bi2Te3 nano-network over a TEM grid after dissolving the alumina membrane. d) and e) Additional 3D Bi_2Te_3 nano-networks with different distances between transversal joints (L), 269 and 580 nm, respectively. Scale bar is 250 nm, in all cases. f) Plot of the difference conductance per transversal channel as a function of the temperature logarithm for 3 different 3D Bi2Te3 nano-networks with L = 269 (green squares), 417 (blue circles) and 580 (red triangles) nm. The y-axis was arbitrarily shifted in the vertical direction to show the three measurements simultaneously. The solid lines represent the linear fit of the data below 10 K, showing the same slope independently of the period, L.

SEM analysis of the samples with and without the 3D AAO membrane confirms that the porous structure of the templates, which is shown in Figure S1a, was successfully filled by the material. Figure S1b shows a cross-sectional image taken after cutting a piece from the original sample. We analyzed different areas from several sample pieces of micrometer sizes at different magnification (see also Fig 1). These images show a controlled and homogeneous growth of the material inside the pores (both vertical and transversal pores). No defects or empty areas were observed. The nanowires present a smooth surface with no discontinuities or defects. After dissolving the alumina matrix in a mixture of 7 wt% H_3PO_4 and 1.8 wt% H_2CrO_4 for 24 hours, and sonication, we can observe a bunch of interconnected nanowires isolated from the 3D nano-network, see figure S1c-e. This image allows us to better appreciate the interconnections between neighboring nanowires (indicated with an arrow), which result from the filling of the transversal nanochannels of the 3D AAO templates (in Figure S1b). However, some nanowires may seem broken or missing due to the sonication step.

Figure S1f shows the resistance at temperatures below 10 K of three 3D Bi_2Te_3 nano-networks fabricated with different periods, 269 (represented in green), 417 (blue) and 580 (red) nm. We systematically observed an upward turn of the

resistance when decreasing the temperature below 10 K. But, more importantly, the slope of the linear fit (solid black line) is the same in the three measured samples, independently of the distance L (or total number of transversal paths, *N*), obtaining similar αp coefficients of 2.0, 1.8 and 2.2 in each case that match the value reported in metallic thin films due to 2-D localization. Then, we suggest that localization effects arise due to the geometry of the nano-networks, in particular due to the transversal joints.

XRD and TEM

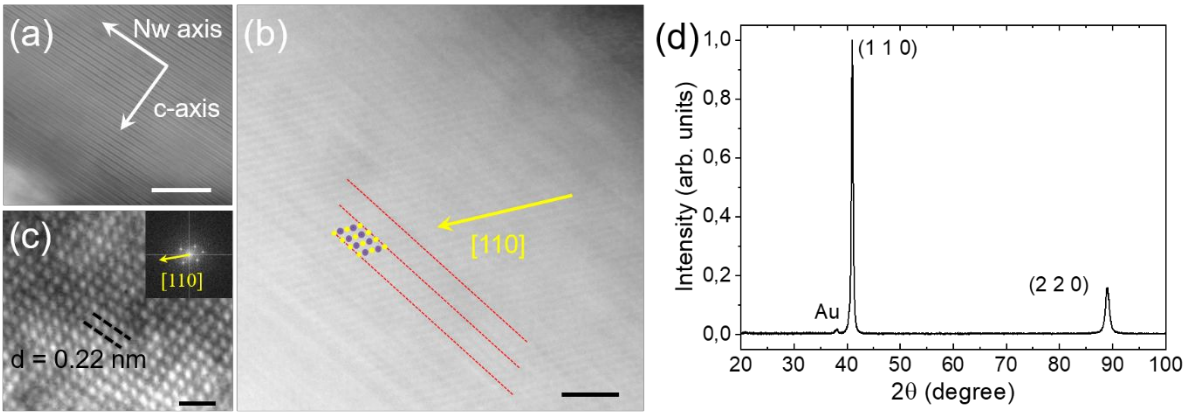

Figure S2. HR-TEM analysis of the dissolved sample: a) Cross-view SEM image of the 3D Bi2Te3 nano-network still embedded in the alumina membrane (as measured), scale bar 10 nm. b) Image along the length of the nanowire of the 3DNN array. b) is an enlarged image of a), in which the red dotted lines indicate the separation between quintuple layers, and the atomic layers of Bi and Te can be distinguished. Scale bar 2 nm c) ⟨0001⟩ zone-axis HRTEM image showing an inter-planar distance of 0.22 nm. The inset image is the corresponding SAED pattern of c), illustrating a hexagonal pattern index to [110] direction, characteristic of single crystals. Scale bar 1 nm. d) XRD diffraction spectra, where only the peaks corresponding to [110] and [200] are found, which means that the c-axis of the Bi2Te3 is oriented perpendicular to the length of the nanowires.

TEM analysis of the dissolved 3D Bi_2Te_3 nano-networks (like the ones shown in Fig S1c) confirmed that the nanowires are highly textured and present a quasi-epitaxial growth along the [110] direction, indicating that the c-axis of the bismuth telluride crystal structure [S1-S2], which is characterized by the presence of Van der Waals forces, is perpendicular to the nanowires' length. This is illustrated in Figure S2a, where the different directions are represented by white arrows. Figure S2b is an enlarged image of Figure S2a where the red dashed lines separate quintuple layers, the yellow arrow follows the growth direction of the Te (in yellow) and Bi (purple balls) atoms, piling up along the nanowire axis. Figure S2c corresponds to ⟨0001⟩ zone-axis high-resolution TEM (HRTEM) image, the value of the inter-planar spacing of the planes, d, was 0.22 nm, which is in good agreement with the d value of the (110) planes of rhombohedral Bi_2Te_3 [S3-S5]. Furthermore, the SAED pattern inset of the area in Figure S2c, shows a hexagonal symmetry diffraction spot pattern, indicating the high crystal quality of the nanowires, that was indexed to the [110] direction. TEM results are in excellent agreement with the obtained XRD diffraction pattern in Figure S2d where the only peaks found correspond to the (110) and (200) direction, which means that the c-axis of the Bi_2Te_3 is oriented perpendicular to the length of the nanowires.

EDAX and RAMAN

The Bi:Te ratio was adjusted to 2:3 by modifying the deposition voltage potential as reported in reference [20] of the main text, to corroborate if we have obtained the desired stoichiometry we measured the composition of the 3DNNs employing different techniques: EDAX and RAMAN.

EDX analysis was performed to determine the Bi and Te percentage in each sample. To obtain reliable results, measurements were taken in different areas along a cross-sectional cut of the 3DNNs still embedded in the alumina matrix. The composition percentages of Bi and Te measured along the axis of the nanowires are shown in Figure S3a on the dissolved nanowires (inset TEM image).

Raman spectroscopy has proven to be a useful tool to identify the presence of certain chemical species as they vibrate (Raman active vibrational modes) when excited. It has been previously employed to study the layered structure of Bi_2Te_3 [S6-S8]. Furthermore, this technique is particularly useful in detecting the presence of Te nano-clusters in Bi_2Te_3 nanowires and nanoparticles [S9-S10].

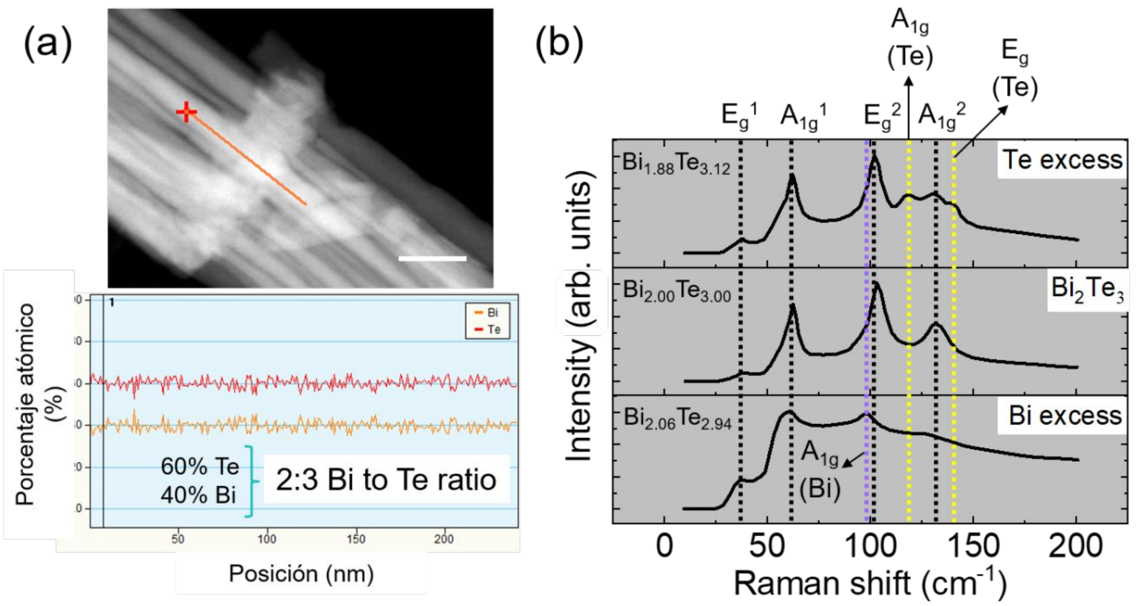

Figure S3. a) EDAX compositional analysis along the nanowires axis. b) Representative Raman spectra of 3DNNs containing Bi excess (bottom), 2:3 Bi to Te ratio (middle), and Te excess (top).

Considering the primitive unit cell of the Bi_2Te_3 crystal layered structure [S1-S2] consisting of 5 atoms in the sequence: - (Te1 – Bi – Te2 – Bi – Te1) -, along the c-axis, there are four active Raman modes, Eg1, A1g1, Eg2, and A1g2, associated to the vibration of the Bi – Te(1 or 2) bonds. Eg and Ag modes are differentiated by the motion of atoms in the direction perpendicular and parallel to the c-axis, respectively. Furthermore, the letter A means that the vibration is singly degenerated, whereas E means that is doubly degenerated. Apart from those distinctions, Eg1, and A1g1 modes, in particular, correspond to the in-phase vibration of the Bi-Te1 bond. Thus, the bond between Bi-Te2 governs the frequency of Eg1 and A1g. In the other two, Eg2 and A1g2, Bi and Te1 atoms move in opposite phases. In such a case, the force of the Bi-Te1 bond will determine the vibration frequency.

In Figure S3b, 3D nano-networks with an excess of Te and Bi are compared to a 3D network with 2:3 (Bi:Te) stoichiometry. In the latter case, four Raman peaks were observed at 37, 63, 103, and 130cm-1, which are related to the four characteristic vibrational modes of Bi2Te3, Eg1, A1g1, Eg2, and A1g2, respectively [S6, S9]. When the sample was prepared with Bi excess, the peak located at 102 cm-1, shifts towards 97 cm-1, corresponding to the Eg vibrational mode of Bi [S10]. The excess of Te in the 3D nano-network was identified by the presence of two extra peaks found at 119 and 140 cm-1 and that were associated with the A1g and Eg vibrational modes of Tellurium [S9, S11]. This technique was implemented when optimizing the deposition conditions as to double-check the chemical composition of the 3D nanowire networks, complementing EDAX analysis.

S1. Chen, Y. L.; Analytis, J. G.; Chu, J. H.; Liu, Z. K.; Mo, S. K.; Qi, X. L.; Zhang, H. J.; Lu, D. H.; Dai, X.; Fang, Z.; Zhang, S. C.; Fisher, I. R.; Hussain, Z.; Shen, Z. X Experimental Realization of a Three-Dimensional Topological Insulator, Bi2Te3. *Science*. **2009**, 325, 178-181.

S2. Fornari, C. I.; Rappl, P. H.; Morelhão, S. L.; Abramof, E. Structural Properties of Bi2Te3 Topological Insulator Thin Films Grown by Molecular Beam Epitaxy on (111) BaF2 Substrates. *Journal of Applied Physics*. **2016**, 119, 165303.

S3. Chen, C. L.; Chen, Y. Y.; Lin, S. J.; Ho, J. C.; Lee, P. C.; Chen, C. D.; Harutyunyan, S. R. Fabrication and Characterization of Electrodeposited Bismuth Telluride Films and Nanowires. *The Journal of Physical Chemistry C*. **2010**, 114**,** 3385-3389.

S4. Lee, J.; Farhangfar, S.; Lee, J.; Cagnon, L.; Scholz, R.; Gösele, U.; Nielsch, K. Tuning the Crystallinity of Thermoelectric Bi2Te3 Nanowire Arrays Grown by Pulsed Electrodeposition. *Nanotechnology*. **2008**, 19, 365701.

S5. Shin, H. S.; Jeon, S. G.; Yu, J.; Kim, Y. S.; Park, H. M.; Song, J. Y. Twin-Driven Thermoelectric Figure-of-Merit Enhancement of Bi 2 Te 3 Nanowires. *Nanoscale*. **2014**, 6, 6158-6165.

S6. Russo, V.; Bailini, A.; Zamboni, M.; Passoni, M.; Conti, C.; Casari, C. S.; Bassi, A. L.; Bottani, C. E. Raman Spectroscopy of Bi-Te Thin Films. *Journal of Raman Spectroscopy: An International Journal for Original Work in all Aspects of Raman Spectroscopy, Including Higher Order Processes, and also Brillouin and Rayleigh Scattering*. **2008**, 39, 205-210.

S7. Richter, W.; Becker; C. R. A Raman and Far-Infrared Investigation of Phonons in the Rhombohedral V2–VI3 Compounds Bi2Te3, Bi2Se3, Sb2Te3 and Bi2 (Te1− xSex) 3 (0< x< 1),(Bi1− ySby) 2Te3 (0< y< 1). *Physica Status Solidi (B)*. **1977**, 84, 619-628.

S8. Zhao, Y.; Luo, X.; Zhang, J.; Wu, J.; Bai, X.; Wang, M.; Jia, J.; Peng, H.; Liu, Z.; Quek, S. Y.; Xiong, Q. Interlayer Vibrational Modes in Few-Quintuple-Layer Bi 2 Te 3 and Bi 2 Se 3 Two-Dimensional Crystals: Raman Spectroscopy and First-Principles Studies. *Physical Review B*. **2014**, 90, 245428.

S9. Rodríguez-Fernández, C.; Manzano, C. V.; Romero, A. H.; Martín, J.; Martín-González, M.; de Lima Jr, M. M.; Cantarero, A. The Fingerprint of Te-Rich and Stoichiometric Bi2Te3 Nanowires by Raman Spectroscopy. *Nanotechnology*. **2016**, 27, 075706.

S10. Mott, D.; Mai, N. T.; Thuy, N. T.; Maeda, Y.; Linh, T. P.; Koyano, M.; Maenosono, S. Bismuth, Antimony and Tellurium Alloy Nanoparticles with Controllable Shape and Composition for Efficient Thermoelectric Devices. *Physica Status Solidi (A)*. **2011**, 208, 52-58.

S11. Torrie, B. H. Raman Spectrum of Tellurium. *Solid State Communications.* **1970**, 8, 1899-1901.
